# Supplementary material for: Infantile Anemia and Iron Treatments Affect the Gut Microbiome of Young Rhesus Monkeys
Source: Microorganisms. 2025 Sep 26;13(10):2256. doi: 10.3390/microorganisms13102256 (PMC12566435; doi:10.3390/microorganisms13102256)
Supplement: Supplementary file 1 [file microorganisms-13-02256-s001.zip › microorganisms-3872208-supplementary.pdf]

Table S1. Nutrient composition of the commercial biscuit diet fed to pregnant and nursing rhesus monkeys to create the postnatal model of infantile anemia.

Ingredients		Minerals		Vitamins	
Protein (%)	15.7	Iron (mg/kg)	225	A (IU/g)	20.0
Carbohydrate (%)	68.9	Zinc (mg/kg)	110	B12 (mg/kg)	0.073
Fat (%)	6.0	Copper (mg/kg)	21	C (mg/kg)	500
Ash (%)	5.3				
Fiber (crude) (%)	4.5				

A standardized diet (5LFD, LabDiet, St. Louis, MO) was fed to adult female rhesus monkeys during the pregnancy and nursing periods to create the postnatal risk model of infantile anemia. Each monkey was provided with 10 biscuits (250 g) per day. Between 20-30% of the infants born to monkeys bred in this way will become iron deficient by 6 months of age due to lower iron reserves at birth and the inability of fulfill iron needs to sustain rapid growth from only breast milk prior to transitioning to solid food. This diet recreates what had been the typical biscuit fed to all macaque colonies prior to 1990, before it was realized that many infant monkeys became iron deficient and the manufacturers of commercial biscuits increased the iron content of the diet up to 400 mg/kg [**30,31**].

Table S2. The iron dextran regimen used to treat anemic infants (IDA) included a vitamin B complex. Each infant rhesus monkey was administered a weekly intramuscular injection (IM, 0.1 mL) of iron dextran (10 mg) along with 0.5 mL (IM) of the following B vitamin complex. Hematological and blood-iron indices of anemic monkeys were typically restored to the normal range within 1-2 months (HgB >120 mg/L and MCV >70 fL).

<u>B-vitamin Complex included with Iron Dextran for the Treatment of Anemia</u>

Vitamin B1	50 mg
Vitamin B2	2.5 mg
Niacinamide	50 mg
Vitamin B6	5 mg
d-Panthenol	5 mg
Vitamin B12	50 mg

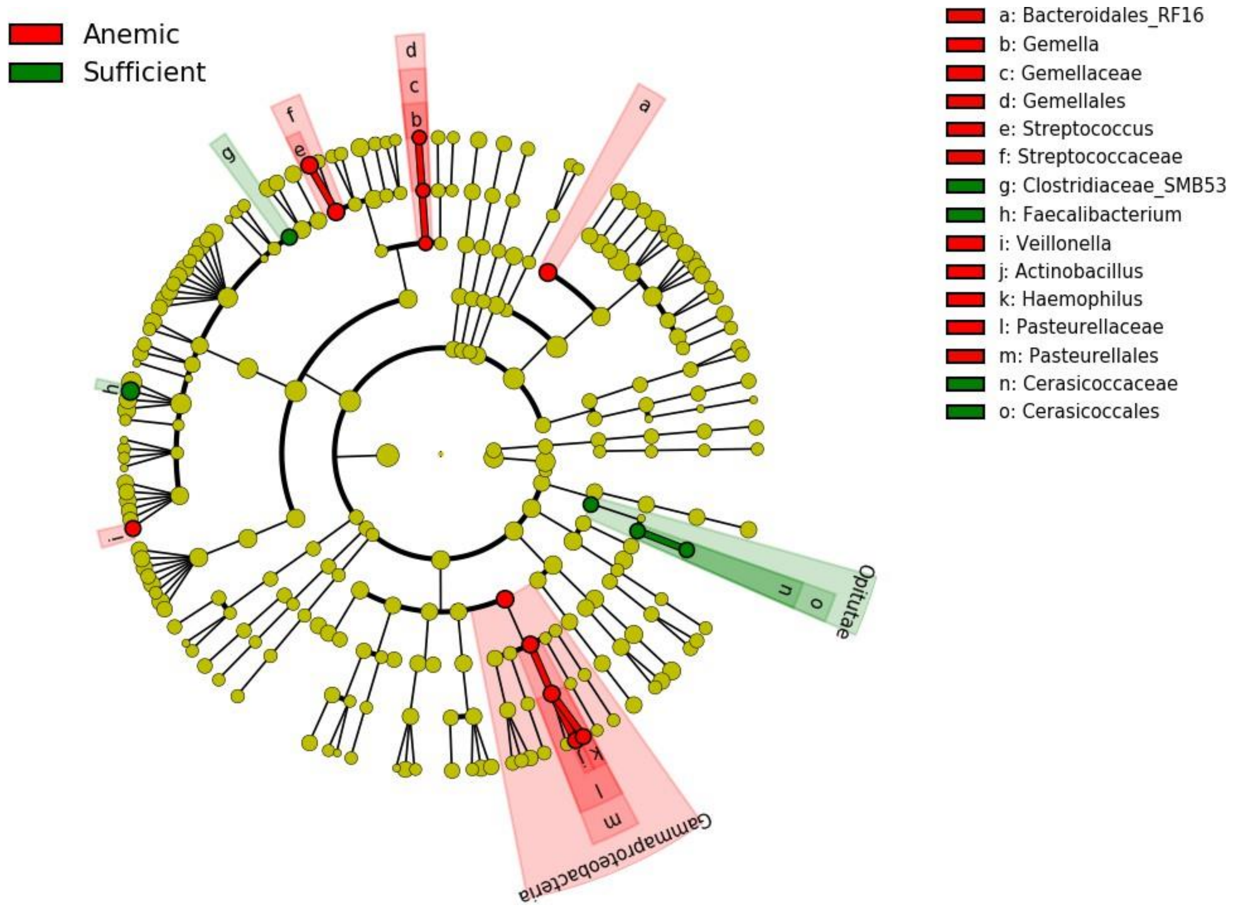

Figure S1. Taxonomic differences between anemic and iron sufficient infant monkey are portrayed in cladogram format illustrating distinctive phylogenetic features by color and height of the vectors. The results from the LEfSe analyses are also illustrated in **Figure 1C**, showing the relative abundances of many taxa with LDA differences that exceeded 2.0.

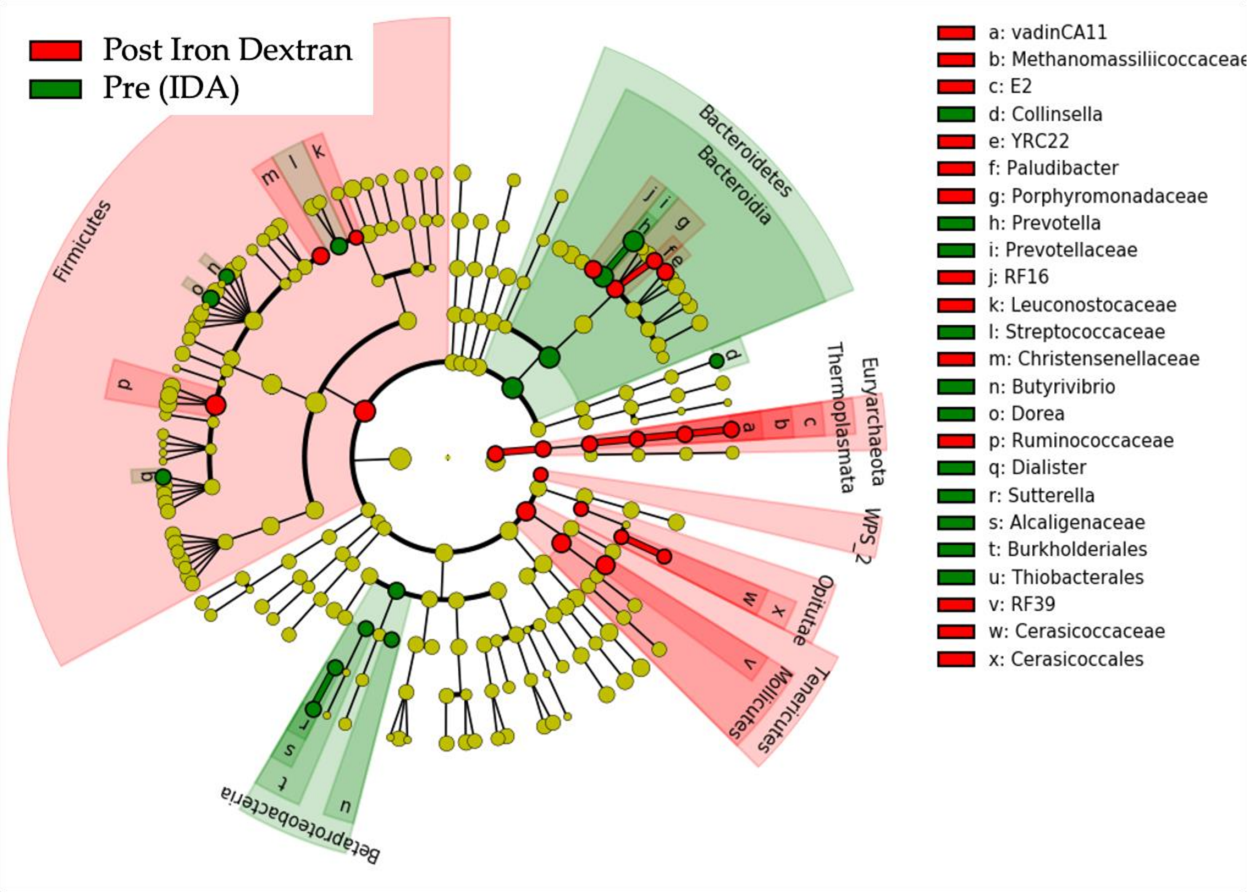

Figure S2. The cladogram shows that the phylogenetic shift at the phylum level after weekly treatment of anemic monkeys with iron dextran (IM) included a relative increase in the abundance of Firmicutes. Prior to treatment, the anemic monkeys had evinced a relatively higher abundance of Bacteroidetes. The results from LEfSe analyses are also shown in **Figure 2C**, portraying the relative abundances of many taxa with LDA differences that exceeded 2.0.

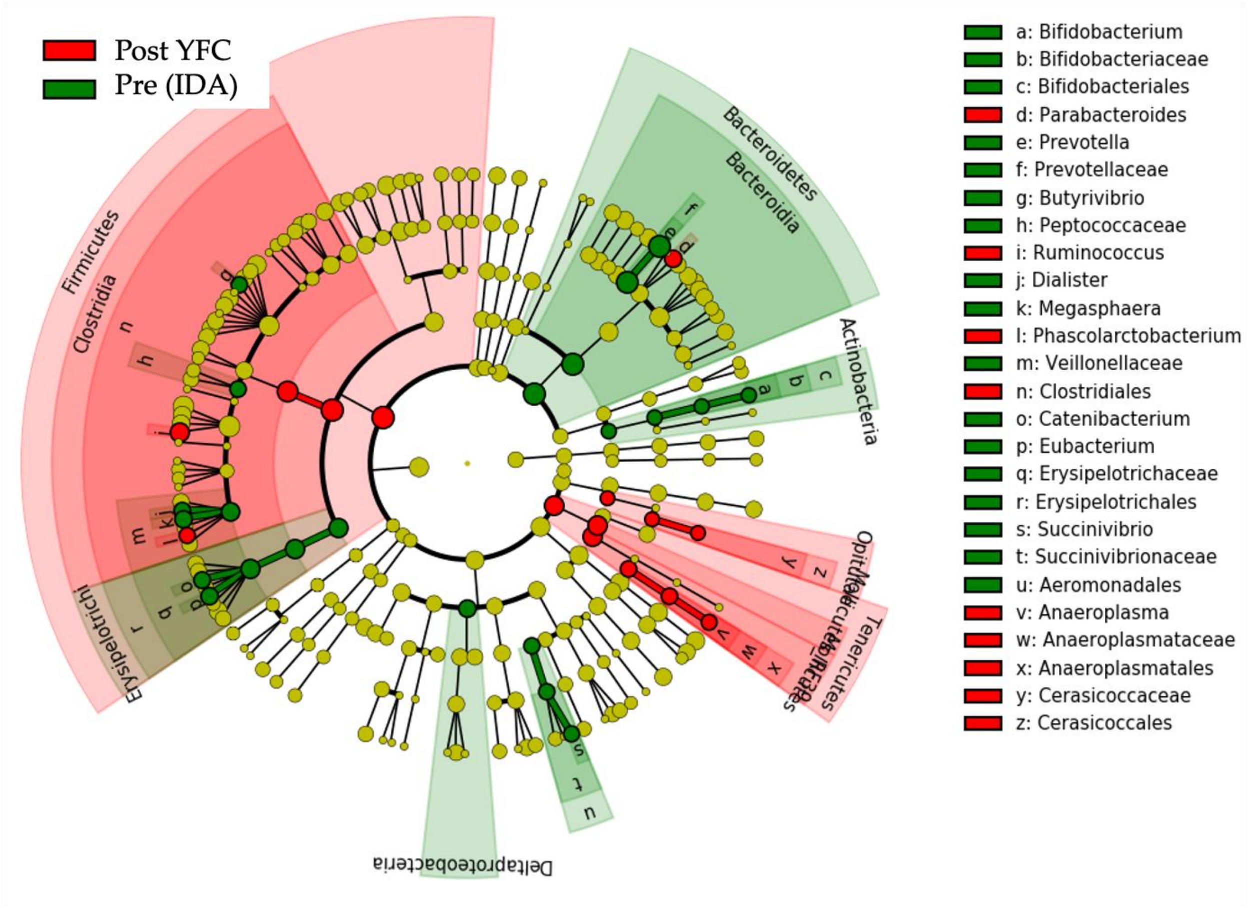

Figure S3. The cladogram illustrates taxonomic changes at the phylum level after oral supplementation of anemic monkeys (IDA) with the yeast-ferritin complex (YFC). The height of the red vectors conveys the relatively increased abundance of Firmicutes and Tenericutes. In contrast, Bacteroidetes and Actinobacteria were more prevalent prior to the treatment of anemic infants. The results from LEfSe analyses are also portrayed in **Figure 3C**, showing the relative abundances of many taxa with LDA differences that exceeded 2.0.

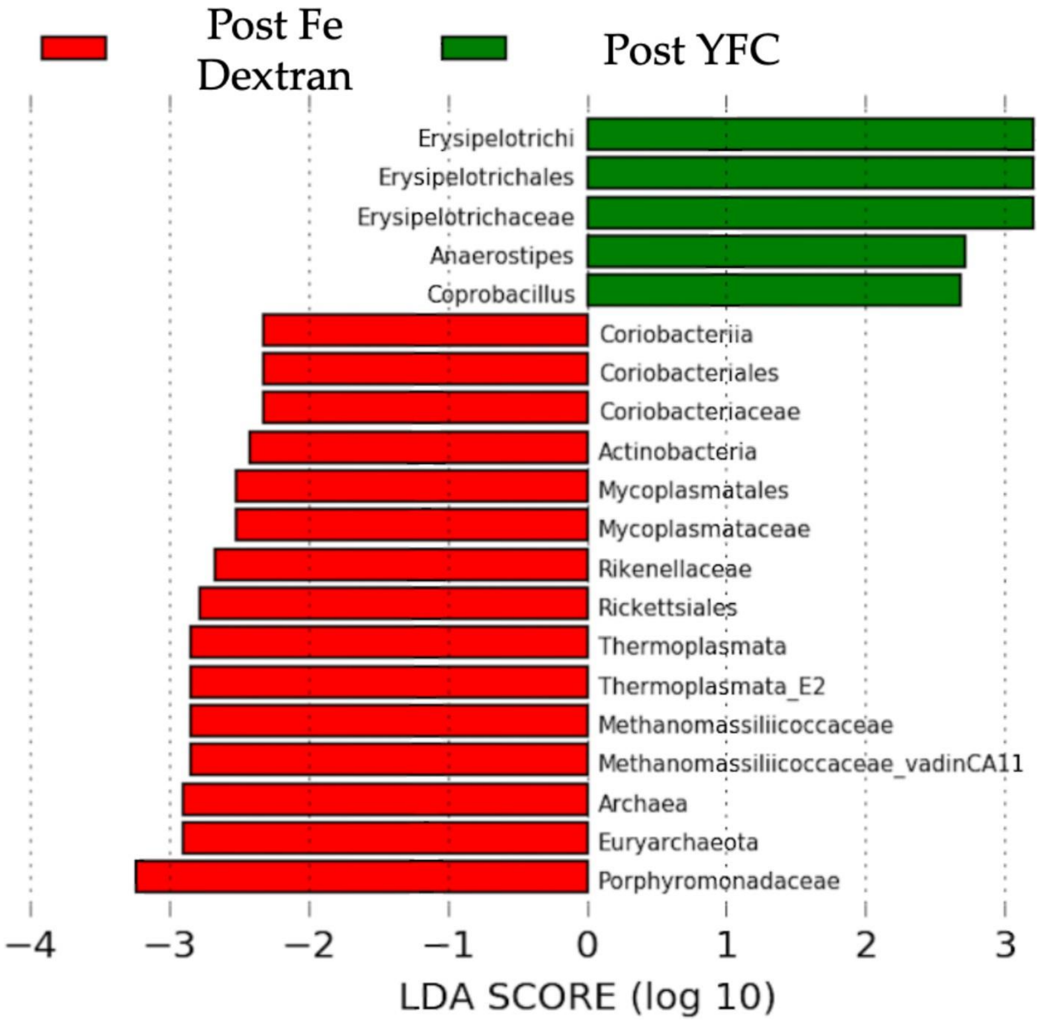

Figure S4. LEfSe analyses indicated that the changes in bacterial composition after treatment of anemic monkeys differed depending on whether it was iron dextran injections or oral supplementation with the yeast-ferritin complex (YFC). In both cases, there were changes from the pre-treatment anemic baseline. The illustration above directly compares the gut microbiome post-treatment in the two conditions. Only taxa with differences that exceeded the threshold LDA score of 2.0 are shown. Seven anemic monkeys were administered iron dextran (10 mg/kg); four anemic monkeys were provided with the YFC orally for 1-2 months (6 mg/kg YFC). The predicted functional pathways inferred as being different after each of the two iron treatments are illustrated separately in **Figures S5** and **S6** based on bacterial genes in taxa with log(LDA) scores that exceeded 2.0.

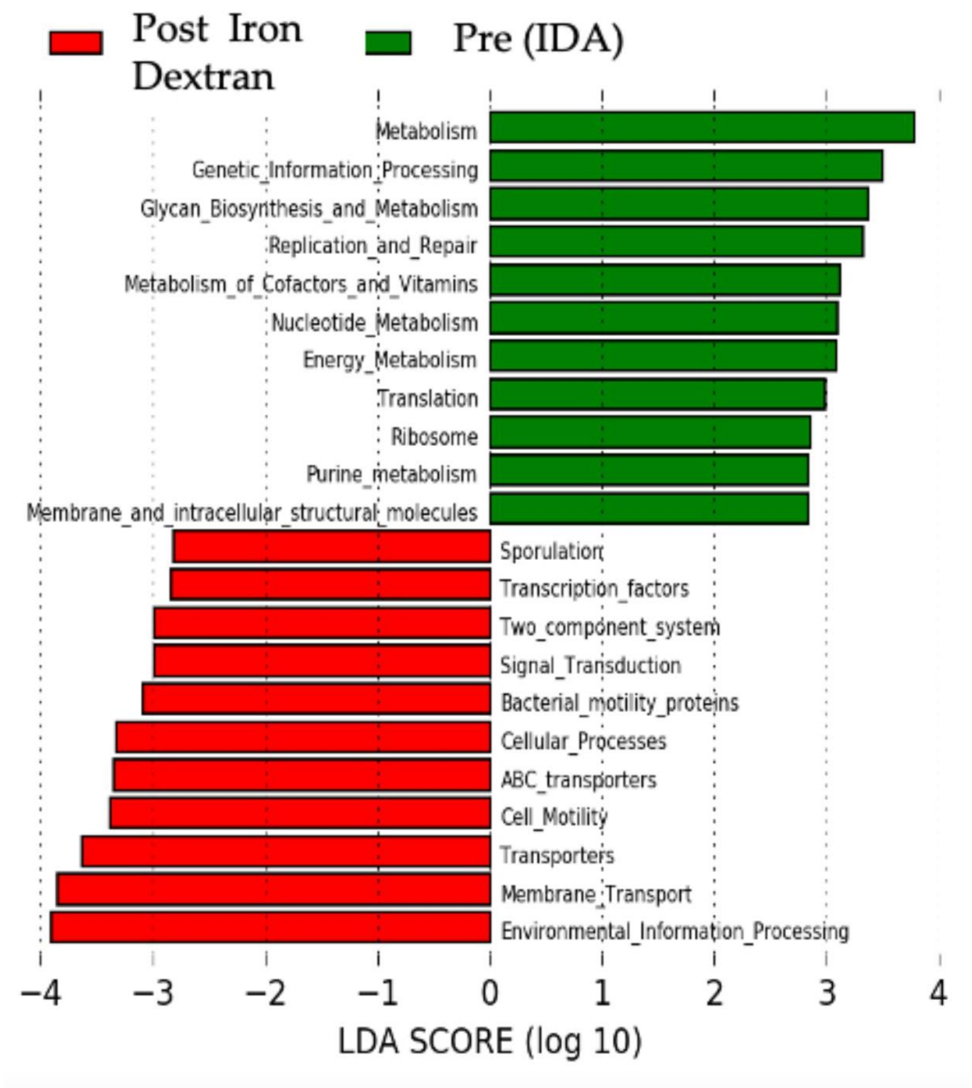

Figure S5. PICRUst analyses were performed to infer possible effects of iron dextran treatment on gut microbial genes associated with functional pathways. Predictions were based on bacterial taxa with LDA scores exceeding 2.0 (see **Figure 2C**). Prior to iron treatment, the gut microbiome of anemic monkeys might exert a stronger influence on several aspects of bacterial and host metabolism, including purine, energy and vitamin metabolism. Post-treatment, many predicted pathway changes were associated with bacterial function, including effects on bacterial and cell motility proteins, and potentially on sporulation, which can be a microbial response to unfavorable conditions.

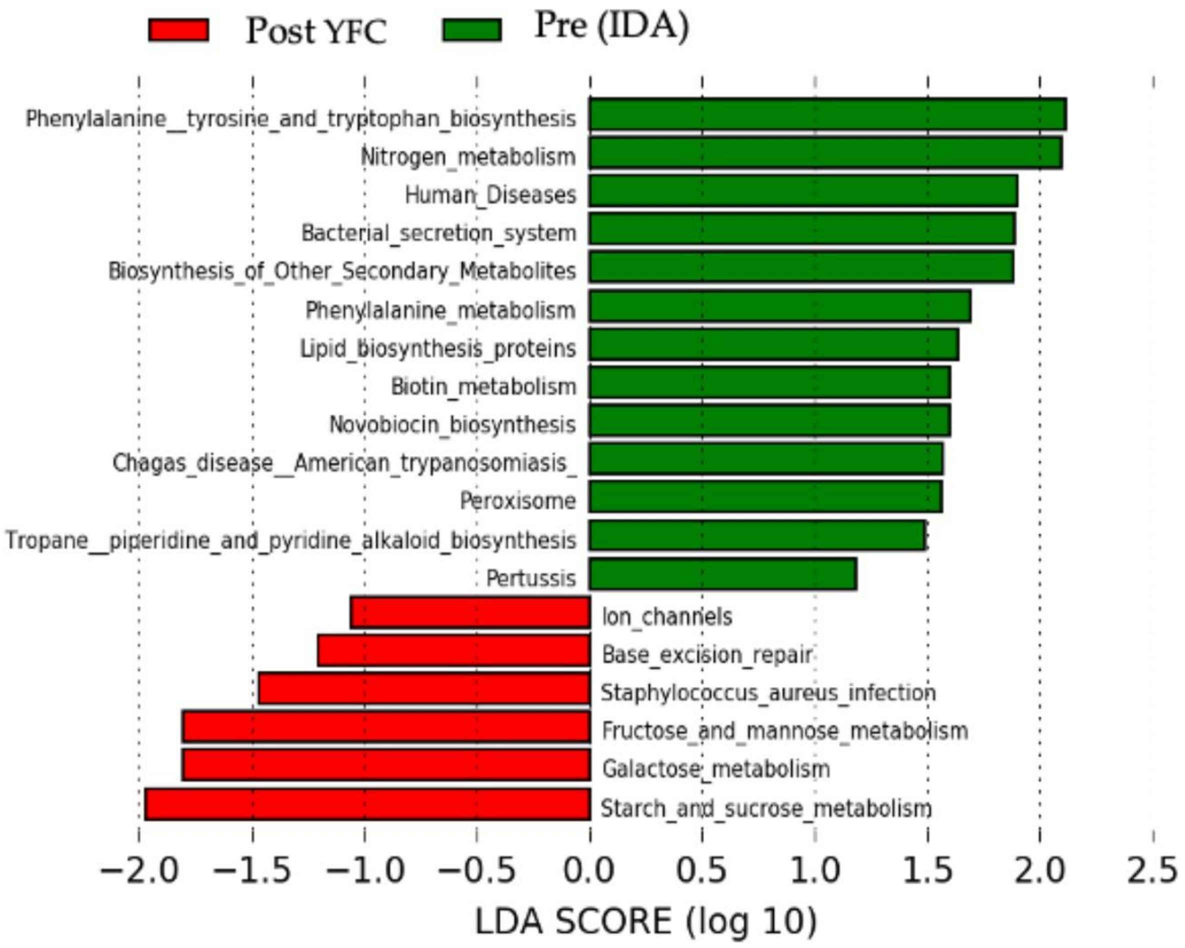

Figure S6. LEfSe testing indicated the relative abundance of many bacterial taxa differed pre- and post-supplementation with the oral yeast-ferritin complex (YFC) (see **Figure 3C**). The potential effects of differences in bacterial genes on predicted functional pathways are shown. Predictions of metabolic effects in the digestive tract, and potentially in the host, are based only on bacterial taxa with LDA scores exceeding 2.0 post-treatment (**Figure 3C**). Prior to treatment, the potential influence of tyrosine and tryptophan biosynthesis in anemic monkeys was notable given the involvement of the gut bacteria in synthesizing precursors to monoamines in the host. During the period of oral ferritin supplementation, the potential effect of microbial genes on pathways associated with fructose, mannose, galactose, starch and sucrose metabolism were more salient.
